# A first glimpse at the transcriptome of *Physarum polycephalum*

**DOI:** 10.1186/1471-2164-9-6

**Published:** 2008-01-07

**Authors:** Gernot Glöckner, Georg Golderer, Gabriele Werner-Felmayer, Sonja Meyer, Wolfgang Marwan

**Affiliations:** 1 Leibniz Institute for Age Research – Fritz Lipmann Institute, Beutenbergstr. 11, D-07745 Jena, Germany; 2 Sektion für Biologische Chemie, Biozentrum der Medizinischen Universität Innsbruck, Fritz-Pregl-Str 3, A-6020 Innsbruck, Austria; 3 Magdeburg Centre for Systems Biology, Otto von Guericke University and Max-Planck-Institut für Dynamik komplexer technischer Systeme, Sandtorstr. 1, D-39106 Magdeburg, Germany

## Abstract

**Background:**

*Physarum polycephalum*, an acellular plasmodial species belongs to the amoebozoa, a major branch in eukaryote evolution. Its complex life cycle and rich cell biology is reflected in more than 2500 publications on various aspects of its biochemistry, developmental biology, cytoskeleton, and cell motility. It now can be genetically manipulated, opening up the possibility of targeted functional analysis in this organism.

**Methods:**

Here we describe a large fraction of the transcribed genes by sequencing a cDNA library from the plasmodial stage of the developmental cycle.

**Results:**

In addition to the genes for the basic metabolism we found an unexpected large number of genes involved in sophisticated signaling networks and identified potential receptors for environmental signals such as light. In accordance with the various developmental options of the plasmodial cell we found that many *P. polycephalum *genes are alternatively spliced. Using 30 donor and 30 acceptor sites we determined the splicing signatures of this species.

Comparisons to various other organisms including *Dictyostelium*, the closest relative, revealed that roughly half of the transcribed genes have no detectable counterpart, thus potentially defining species specific adaptations. On the other hand, we found highly conserved proteins, which are maintained in the metazoan lineage, but absent in *D. discoideum *or plants. These genes arose possibly in the last common ancestor of Amoebozoa and Metazoa but were lost in *D. discoideum*.

**Conclusion:**

This work provides an analysis of up to half of the protein coding genes of *Physarum polycephalum*. The definition of splice motifs together with the description of alternatively spliced genes will provide a valuable resource for the ongoing genome project.

## Background

Analysis of cellular states under well-defined conditions can be performed on different levels. One important method is the generation of expressed sequence tags (ESTs), short informative sequences generated from mRNAs of the cell. The ESTs provide a snapshot of the transcriptome and the conditioned capabilities in the investigated cell state. Moreover, ESTs are a resource for the annotation and further analysis of genome sequences in the respective organism, since they identify transcribed loci within the genome. Comparisons to genome or EST sequences of related organisms are then used to elucidate the functional capabilities of an evolutionary branch.

Many genomes and transcriptomes are currently characterized and more will follow in the future [1]. Typically, only key species in each branch, so-called model organisms, are submitted to a full genome analysis and further studies from a global point of view [2]. Initially, model organisms were chosen according to the following criteria: i) being the best studied species on an evolutionary branch ii) having a short life cycle, iii) being cost effective to maintain, iv)having the potential to be manipulated. In the animal kingdom prominent models include *Drosophila *for insects, *C. elegans *for primitive metazoans, and mouse for mammals. Accordingly, in the plant and fungi kingdom there are also well defined model organisms [3,4]. Yet, model organisms can not fully describe and represent a whole evolutionary branch. Since models are real species and therefore have suffered from specializing adaptations for their specific ecological niche, more than one model within one evolutionary branch should be taken in account [5,6]. Furthermore, the more genomes that are known in a certain evolutionary branch, the more is known of the branch characteristics. Thus, the comparison of multiple species within an evolutionary branch is becoming more and more common. Helpfully, analysis of each subsequent genome within the same evolutionary branch is easier. Currently the best-surveyed eukaryote kingdom of life from a genomics perspective is that of the fungi [4]. Metazoa and green plants are also represented with several complete or nearly completely sequenced genomes [1].

Protists as a non-taxonomic unit have great genomic and phenotypic diversity. The position of specific protist branches in the phylogenetic tree of life is often unsure, if not controversial. Complete genome sequences can help to resolve even deeply branching phyla. From one branch of protists, the amoebozoa, we currently have full sequence information of two species, namely *Dictyostelium discoideum *[7] and *Entamoeba histolytica *[8]. This branch is now generally accepted as a major kingdom within the eukaryotes in addition to fungi, plants, and animals [7,9,10]. While *D. discoideum *possesses the main aspects of a model organism, obligate parasites like *E. histolytica *are of limited use for the intended description of evolutionary trends, since they very often have lost genetic information due to their adaptation to a host. Thus, the full width of this evolutionary branch can only be described if free living, relatively distantly related organisms, are compared. The free living amoebozoon *Physarum polycephalum *is best suited to characterize the functional capacities of this evolutionary branch further. Currently a project targeting the whole genome of this organism is underway at the Genome Sequencing Center of Washington University [11]. We complemented this approach by generating expressed sequence tags (ESTs) from protein-encoding loci of this species. Here we describe the analysis of these ESTs.

*P. polycephalum *SCHWEINITZ is an acellular slime mould (*Myxomycetales*) with a complex life cycle. Under favorable conditions, mononucleate haploid *Physarum *amoebae hatch from mature spores. These myxamoebae feed on bacteria and multiply by cell division with an open mitosis during which the nuclear envelope disaggregates [12]. At high population density two amoebae of compatible mating types may mate to form a mononucleate diploid zygote [13]. The zygote develops into a diploid multinucleate plasmodium since the plasmodial cell does not divide, while nuclear divisions continually occur as the cell grows. Closed mitosis by division of plasmodial nuclei is often found in protists [14] while open mitosis as it occurs in the amoebal stage is typical for higher eukaryotes. A plasmodium grows as long as nutrition is available, resulting in a macroscopic mass containing millions of nuclei which all are synchronous with respect to cell cycle and development [15]. Despite their unlimited capability for growth and nuclear division, plasmodia can enter two alternative developmental pathways (spherulation or sporulation) depending on environmental conditions and cell size [15]. When a big mature plasmodium (macro-plasmodium) starves, it develops the competence to sporulate. Sporulation of a competent plasmodium is finally triggered by visible light and/or elevated temperature ([15-17], and references therein). During sporulation the entire protoplasmic mass develops into fruiting bodies. In sporangia, haploid mononucleate spores are formed by meiotic cleavage of the plasmodial mass.

For our analysis of the transcriptome of *P. polycephalum *we used this sporulation- competent state. Since the cells in this state still have all options of growth and development, we expected that vegetative specific genes would be only slightly reduced and that additional factors needed to progress to the commitment point would also be expressed. Using this method we can find more genes than either in the vegetative or committed state.

## Results

### Generation and clustering of ESTs

In total 15,680 directionally cloned cDNAs were sequenced from the 5' to the 3' end of the corresponding mRNA resulting in 5856 contigs after clustering (Table 1; accession numbers [dbEST: EL563888 to EL579567]). Since the sequences were all generated from the 5' end of the corresponding mRNA and the library was enriched for long inserts, each contig should represent a single gene. Transcripts from very recent gene duplications could not be discriminated if no single nucleotide polymorphisms (SNPs) were present in overlapping regions. The mean coverage of each contig is 2.4 with less than half of the contigs represented by only one sequence read. The most abundant transcript in the library codes for a non-transporter ABC protein of which the exact function is not known. The relatively low sequencing depth shows that the normalization procedure was successful.

**Table 1:** Statistics of the cDNA sequencing and clustering

total sequences	15,680
total bases analysed	10,582,089
average length of reads (bp)	674.88
number of contigs	5,856
number of contigs consisting of a single read	2,787
total contig length (bp)	4,425,063
average length of contigs (bp)	755.6
longest contig (bp)	2,581
highest number of ESTs in a contig	96

### Similarity of EST contigs to other organisms

Based on BLAST analysis, 3282 of the 5856 potential genes had detectable similarities to entries of a database containing all currently available sequences (TrEMBL). Obviously, not all similarities found must indicate true orthologous relations, since the EST information does not always cover the whole gene. Yet, due to the partial nature of the gene information in EST data it is also not feasible to introduce a score cutoff to get only reliable information. The closest free-living relative, of which the whole genome is known, is *D. discoideum*. We conducted a search for similarities to protein coding genes in this genome and found that 2387 of our contigs matched at least partially to *D. discoideum *proteins indicating a relationship between the matching pairs. Surprisingly, we found 895 contigs with similarities to entries in other databases but not in the *D. discoideum *protein database. This can be partly due to false positive matches, but some identified proteins hint at a scenario where *D. discoideum *has lost the respective gene as was shown previously for some genes (see below) [18].

We also analysed, which evolutionary branches contained genes with similarities to our contig sequences. For this purpose we used sequence databases representing only certain parts of the evolutionary tree (see Materials and Methods). As with the screen in the complete sequence database TrEMBL we initially did not limit the BLAST output by using a restricting threshold. Matches were then assigned to subgroups to give information on the phylogenetic distribution of the best matching proteins. In Table 2 the results are compiled for the listed categories. Since spurious hits may limit the value of the analysis, we introduced a score threshold of 150 in a second round of analysis (Table 2; numbers in brackets). The number of contigs without any hit to known genes increases slightly when we use this threshold, but the overall distribution of database matches remains the same, indicating that the values given are generally applicable.

**Table 2:** Blastx matches to various databases and alternatively spliced transcripts in each category. Numbers in parenthesis are calculated using a blastx threshold score of 150.

Category		Number of contigs	more than 1 read	alternatively spliced	% of contigs (no singletons)
All groups		960 (640)	629 (439)	105 (75)	16.7 (17.1)
Eukaryote					
	All (sum)	1947 (1607)	1139 (1012)	189 (168)	16.6 (16.6)
	All Eukaryotes	511 (420)	309 (265)	47 (38)	15.2 (14.3)
	Amoebozoa/Protozoa	370 (323)	210 (199)	38 (29)	18.1 (14.6)
	not plant	513 (415)	281 (259)	52 (53)	18.5 (20.5)
	not vertebrate	87 (60)	50 (37)	7 (5)	14.0 (13.5)
	not plant/vertebrate	58 (54)	39 (33)	8 (6)	20.5 (18.2)
	plant	204 (182)	129 (120)	18 (22)	14.0 (18.3)
	vertebrate	204 (153)	121 (99)	19 (15)	15.7 (15.2)
Prokaryote		237 (209)	109 (102)	32 (20)	29.4 (19.6)
Physarum specific		2451 (3166)	1045 (1383)	179 (243)	17.1 (17.6)
not defined		261 (234)	147 (133)	24 (23)	16.3 (17.3)
sum		5856	3069	529	17.3

Next, we searched for genes, which are present in vertebrates, but cannot be detected in *D. discoideum*, invertebrates, or plants. Presumably, these proteins evolved before the split of the the amoebozoa from the eukaryote/fungi kingdoms but were lost individually in invertebrates and in *D. discoideum*. In Table 3 and Additional file 1, the best matching contigs to vertebrate database entries can be found.

**Table 3:** *P. polycephalum *genes with high scoring matches on the Metazoa lineage without matches to *D. discoideum *or invertebrate proteins as identified by similarity search against Swissprot

Contig identifier	Database description of best Hit	Blast Score	e-value
EL565900	(Q8BVC4) similar to CTCL Tumor antigen SE57-1	635	2.50E-087
EL565233	(Q5T3U1) EGF-like-domain protein	628	1.00E-059
EL579005	(Q54K67) Alpha-mannosidase	584	4.10E-054
EL568255	(Q6NTQ6) MGC83094 protein	543	1.00E-050
EL566750	(Q86AV9) Phosphatidylinositol-glycan-specific phospholipase D 2	512	1.50E-046
EL566856	(Q54HV5) hypothetical protein	486	1.10E-044
EL565034	(Q9D945) hypothetical protein	460	6.40E-042
EL565184	(Q1HFZ0) Myc-induced SUN domain containing protein	455	9.10E-041
EL565654	(Q3SYX2) hypothetical protein	355	1.80E-030
EL574525	(Q8R432) Glycosylphosphatidylinositol phospholipase D	307	1.30E-024

### Domain distributions, pathway analysis, and signal transduction

The Kyoto Enzyclopedia of Genes and Genomes (KEGG) is a valuable resource to evaluate the presence or absence of pathways. Despite the incomplete nature of EST sequence data, we were able to identify most components of the basic metabolism by using the KAAS server from the KEGG database. For example, we found the transcripts of 19 aminoacyl-tRNA synthetases. Two transcripts for phenylalanyl-tRNA synthetase are present, while the transcripts for histidyl- and valinyl-tRNA synthetase are missing in our data set. Furthermore, the transcription and translation machineries as well as the replication and repair components are nearly completely represented in our dataset. This is consistent with the previous finding that protein synthesis and nuclear divisions still occur in the starving, competent plasmodium, although the cytoplasmic mass continually decreases [19]. Certainly, protein synthesis depends on intact metabolic pathways. Except for malate dehydrogenase, the enzymes of the citric acid cycle were represented at the RNA level.

Many components of the wnt, MAPK, notch, Calcium, and phosphatidylinositol signaling pathways are present in our data set. Also represented were the transcripts of genes involved in the regulation of the cytoskeleton or cell cycle progression [see Additional file 2].

To check whether *P. polycephalum *uses additional main pathways compared to *D. discoideum *we queried the KEGG database again using only those contigs without a match to *D. discoideum *genes. All contigs classified by KEGG also have a *D. discoideum *counterpart (not shown).

**Figure 1:**
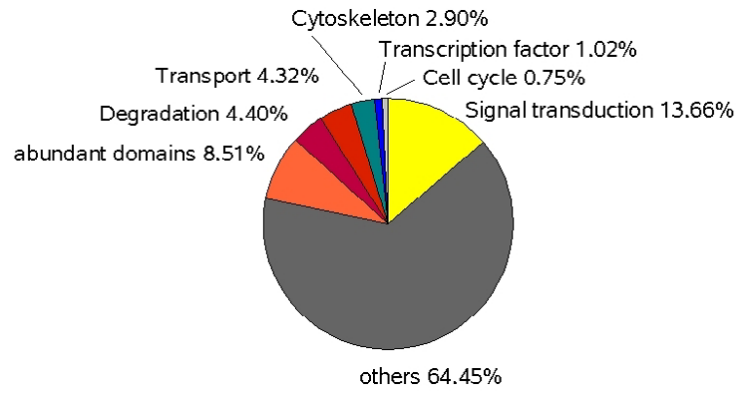
Distribution of main categories in all contigs based on IPR domains.

The scan against Interpro domains yielded 11091 potential domains in all 6 frames in the raw output [see Additional file 3]. A total of 2914 contigs contain at least a part of an Interpro domain [see Additional file 1]. To avoid redundancies caused by potential sequencing errors resulting in frame shifts this raw list was then reduced to 4492 entries by only counting one frame. Visual inspection showed that in most cases the additional hits on other frames were the same as in the counted frame indicating sequencing errors that caused a frame shift. The remaining entries were grouped according to their involvement in different cellular processes (Fig. 1). The sizeable proportion of genes expressed for the generation and maintenance of signal transduction chains indicates that this seems to be a major task of the starving plasmodium. In addition, genes for transport processes and the cytoskeleton play a significant role at least under these conditions.

We found 490 contigs that had no similarity to any database searched using BLAST but contained an Interpro domain. This number nearly doubles (829) if we apply a threshold score of 150. Not surprisingly most of the detected domains are relatively short domains involved in protein-protein interactions.

**Figure 2:**
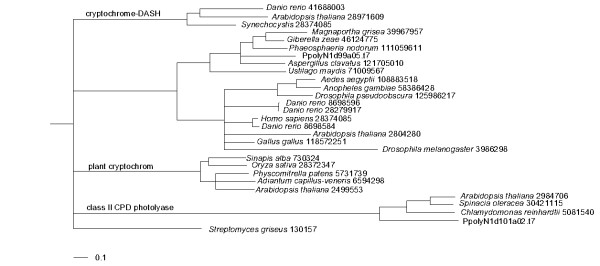
Phylogenetic position of detected photolyase genes. Numbers after the species description denote GenBank accession numbers. The tree was calculated using the neighbor joining method with the phylip package.

The physiological state from which the mRNA for library construction was obtained can be described as a stand-by position where the cell waits for environmental input. We therefore searched specifically for domains forming receptor structures or involved in receptor functions. In 27 of our contigs we found these domains (Table 4). Since receptors are often present in genomes as large families [20-22] the specific trigger for a certain receptor molecule cannot be inferred from sequence analysis alone. Three contigs encode proteins of the photolyase family. All photolyases sense light and some family members are known to function as receptors for certain light qualities [23,24]. One of these potential receptors [dbEST:EL577394] is most closely related to fungal proteins, but the function of these proteins is still unknown (Fig 2). The other two family members cluster together with class II CPD photolyases, which are involved in UV damage repair processes [25]. Possibly, one of these light sensing molecules is the activator of a signal transduction chain inducing sporulation.

**Table 4:** Putative receptors expressed during the transition state of *P. polycephalum*

description	IPR ID	contig ID (dbEST number of first EST in contig)
Rhodopsin-like GPCR superfamily	IPR000276	EL567627; EL568021; EL566982; EL573085; EL577787; EL564481; EL566085
GPCR, family 3, metabotropic glutamate receptor-like	IPR000337	EL574348; EL568458; EL572609; EL576960; EL567878
Recoverin	IPR001125	EL567776; EL565420; EL564706
Opsin	IPR001760	EL575032; EL569730
Extracellular ligand-binding receptor	IPR001828	EL567677; EL577279
Transient receptor potential protein	IPR002153	EL564693
Cell surface receptor IPT/TIG	IPR002909	EL565288; EL568665
DNA photolyase, N-terminal	IPR006050	EL567047; EL577394
ERG2 and sigma1 receptor-like protein	IPR006716	EL565649
Opioid growth factor receptor (OGFr) conserved region	IPR006757	EL566009
DNA photolyase, FAD-binding, C-terminal	IPR008149	EL577955

### Alternative splicing

The clustering of the single ESTs to contigs revealed that there were many sequences with identical sections but that could not be put together in one contig without introducing gaps in the alignment. We concluded that the corresponding mRNAs were transcribed from the same locus on the genome but differentially processed. This alternative splicing of transcripts is a common feature for all Eukaryotes, but the extent of transcript diversification varies between species [26]. We determined the number of alternatively spliced loci by clustering the ESTs irrespective of whether gaps were created in the alignment. Example alignments of alternatively spliced ESTs are shown in additional file 4. With this method 529 potential alternatively spliced messages were constructed comprising 9 % of all clusters. Further analysis revealed that alternative splicing affects genes regardless of their phylogenetic similarities, since no significant differences were found between phylogenetic categories using the χ^2 ^test. We tried to find genes, which can be alternatively spliced in *P. polycephalum *as well as in *D. discoideum*, the closest relative. This is perhaps unlikely as there are only 18 known alternatively spliced genes in *D. discoideum *[27]. A comparison with these genes revealed no gene alternatively spliced in both organisms.

Several alternatively spliced transcripts were of the intron retention type as could be deduced from potential splice sites at the border of the longer alternative transcript. Those introns are normally relatively short and rare in humans but widespread in Arabidopsis [28,29]. We tried to resolve the genomic structure of some of the alternatively spliced transcripts. For this purpose we chose 25 alternatively spliced contigs randomly and designed primers for an amplification of the known part of the genes from genomic DNA. Nine out of 25 amplification attempts were successful, i.e. gave products, which could be subsequently sequenced. The low success rate is possibly due to wrong placement of oligos for PCR on the EST sequences since we did not know whether and where introns were present in the gene loci. The alignment of the resulting genomic sequences to their corresponding transcript counterparts revealed that all ESTs were correctly clustered despite the differential splicing. We could determine all donors and acceptors from 32 introns [see Additional file 5]. Of these 32 introns, 13 introns were not spliced out in a part of the ESTs, thus comprising the number of intron retentions via alternative splicing. Nine introns had alternative donors or acceptors, and 10 introns were not affected by alternative splicing, i.e. all ESTs lost the respective intron. In one case we identified a small additional exon in a transcript, which otherwise is considered as intron part and skipped. The longest defined spliced intron comprises 994 bases. Interestingly, 935 bases of this intron are A or G nucleotides (94 %), whereas in the second largest intron in this gene 92 % are C and T nucleotides [see Additional file 5]. Thus, intron base composition can be very diverse even in only one gene. Two of the fully spliced introns represented U12 dependent introns, thus confirming that this type of intron is present in all evolutionary branches.

**Figure 3:**
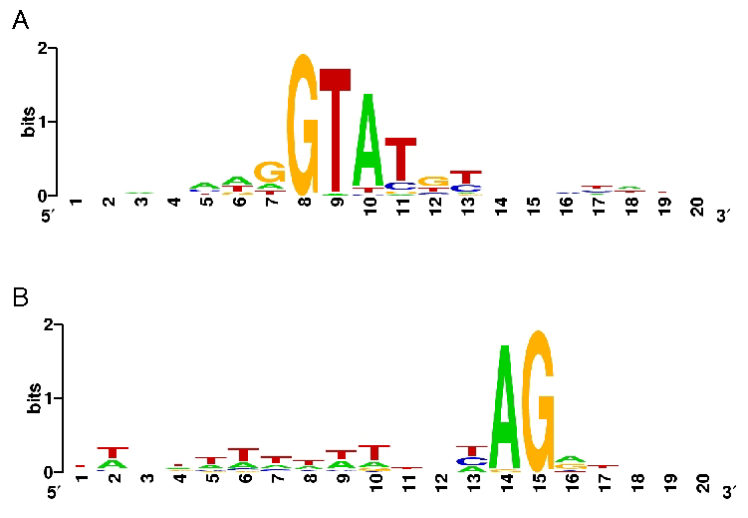
Intron signatures derived from 30 splice donors (A) and 30 splice acceptors (B). The signatures were calculated using Weblogo [30].

We aligned all our U2 dependent donor and acceptor sites (30 each) to calculate the consensus splice motifs. In Fig. 3, donor and acceptor consensi are shown as weighted base probabilities [30]. Interestingly, as in *D. discoideum*, the splice site consensi do not extend far from the canonical GT-AG motif, neither into intronic nor exonic regions, contrary to splice site motifs in animals and plants. A slight preference for pyrimidines can be found at the acceptor site, and at the intron side of the donor, A and T are preferred at the second and third position after the splice site. This resembles the situation in *D. discoideum*.

## Discussion

### Genomic differences within the Amoebozoa

One of our goals was to investigate the common capabilities within the amoebozoan evolutionary branch, and 41 % of all contigs could be assigned to related proteins of *D. discoideum*. That this matching percentage is not higher might be due to several different possible reasons: i) EST contigs represent only a fraction of the whole gene ii) *D. discoideum *has a high nucleotide bias that also influences amino acid usage possibly obscuring protein-protein relationships iii) The evolutionary distance of the two species is large, thus evolutionary specializations account for the observed differences.

More surprising is the observation of nearly 1000 contigs with more or less conserved counterparts in other genomes but not *D. discoideum*. These genes are not involved in basic pathways as the KEGG Pathway analysis showed. The corresponding genes could have been lost in *D. discoideum *since the separation of the two species. Another possibility is that these genes evolved faster in *D. discoideum *than in other species (see above).

### Functional categories and pathway analysis

The starving, competent plasmodium is in an undetermined physiological state in that it has three options of development: re-enter growth, spherulate or sporulate. When a competent plasmodium finds a nutrient source, it re-enters growth, when it is submersed by water, it spherulates or when it is induced by light or heat shock, it sporulates [15].

Therefore, a starving, competent plasmodium is in a delicate limbo state primed for several options. This state is destabilized if receptors and signal transduction chains receive environmental signals. We choose this state for our analysis, since the cell should generate mRNAs for all its possible future fates. As a result, it is not surprising that we could find major signal transduction chains. Yet, what was unexpected was the percentage of all contigs they represented indicating the presence of a sophisticated signaling network. We also could define potential receptors, which, upon activation, could be responsible for the determination of the plasmodial fate (Table 4). One of the potential light receptors [dbEST:EL577394] obviously belongs to a family of well conserved proteins, but the functions of proteins in this specific family tree branch remain to be determined.

The main metabolic and housekeeping genes are still expressed in this state. This indicates that at this point in the life cycle of *P. polycephalum*, the normal turnover of material can take place and this might be responsible for its ability to resume growth instead of committing to spherulation or sporulation. Whether the transcription and translation rate is reduced in this specific state remains to be determined. Since the starving, competent plasmodium has other choices depending on the environmental signals received, these genes involved in this simple form of cell fate specification are also expected to be expressed in the plasmodium. However, the exact nature of them can not be determined from the sequence alone. Further functional studies have to be performed to define these components.

### Alternative splicing and intron signatures

Levels of alternative splicing differ between organisms. The best analyzed species are vertebrates, where alternative splicing is thought to contribute to transcriptional and, consequently, organismal complexity. Yet, even within vertebrates there are large differences in levels of alternative splicing [26]. Moreover, alternative splicing is not well conserved on the gene level, i.e. closely related species can differ in their splicing patterns in certain genes [31,32]. *P. polycephalum *seems to possess, compared to other lower eukaryotes like dinoflagelates and diatoms, extensive alternative splicing capacities (own observations). If we compare the nearly 10 percent alternative splicing events with the only 19 described alternative transcripts in *D. discoideum*, we find no overlap of alternatively spliced transcripts in the two amoebozoa. Since the genome of *D. discoideum *is with 34 Mb a factor of 10 smaller than that of *P. polycephalum *with ca. 240 to 300 Mb [33,34], one is tempted to conclude, that the amount of alternative splicing could be partly positively correlated to genome size.

The longest intron we detected consists almost exclusively of A and G nucleotides. If many such introns exist in the genome, we expect that the assembly of whole genome shotgun sequences will prove difficult in these regions.

### ESTs as a resource for a whole genome project

Lower single-celled eukaryotes contain between 6 and around 13 thousand protein coding genes as currently available data show [7,35,36]. The only available species related to *P. polycephalum*, *D. discoideum*, was predicted to contain around 13,000 genes [7]. The largest protein coding gene repertoire known is currently that of *Paramecium tetraurelia *with around 40,000 genes, yet this high number is due to a recent genome duplication [37]. Even human genomes do not encode for more than around 20,000 genes [38]. Thus, the number of protein coding genes in *P. polycephalum *should be in the same range. We think that with the nearly 6,000 EST contigs we described parts of at least 30 % of all genes in *P. polycephalum*. Sequencing of significantly more ESTs would increase the amount of data only slightly. Accordingly, the remaining 50 % of genes are expected to be either specifically expressed during other stages of the life cycle or only weakly or both. This conclusion is supported by the fact that the much deeper coverage with ESTs (> 150,000; [27]) of the *D. discoideum *genome was not sufficient to cover most of the genes. Even this amount of EST data covers less than 70 % of all predicted genes in *D. discoideum*. Thus we think that we have found a large fraction of the genes, which can be defined using EST data. For the ongoing genome project for the *P. polycephalum *genome this is a valuable resource. It can be used to define transcribed loci even if no similar counterparts are available in the databases. Additionally, the splice site patterns found here can be used for the training of gene finding programs so that the automated gene prediction can be more reliable. Our finding, that a considerable part of the transcripts is alternatively spliced pinpoints the additional problems which could be associated with the gene finding process.

## Conclusion

We provide an extensive overview on the transcriptional activities of a sophisticated acellular system, *Physarum polycephalum *in a well defined multi-potential state. The presence of the primary metabolism demonstrates its not yet committed state, whereas the presence of various signal transduction chains points at its alertness towards possible environmental changes. The surprisingly high extent of alternative splicing we observed could reflect an additional power to adapt to changing environments via slightly changed transcripts.

## Methods

### Strain and growth of plasmodial cells

Plasmodia of the white strain LU897 × LU898 were hatched from spherules, grown as micro-plasmodial suspension in liquid shaken culture and the plasmodial mass was then applied as a ring on 9 cm diameter starvation agar plates as described [39]. Micro-plasmodia (ca. 1 g fresh weight on each plate) spontaneously fused to give a single plasmodium in each plate. Plasmodia were starved for six days to obtain maximal competence for sporulation. To verify the sporulation-competent physiological state, plasmodia were cut into two halves. One half was immediately frozen in liquid nitrogen for subsequent extraction of RNA. The other half was returned to the dark and incubated until the next day to verify that the plasmodium had not been induced to sporulation. Each plasmodium was frozen separately to prevent a potential contamination of the sample with plasmodia that were induced or committed to sporulation. The sporulation-competence of the batch was verified by a control in which a certain number of the plasmodia were irradiated for 30 Min with far-red light [40] but otherwise treated identically. None (0%; n = 98) of the non-irradiated plasmodia and all (100%; n = 66) of the irradiated plasmodia sporulated. This indicates that the sample was in a homogeneous physiological state and that the analysed RNA pool was derived from plasmodial cells that were highly competent for sporulation but were neither induced nor committed to sporulation.

### Library construction and sequencing

Total RNA was isolated from frozen plasmodia using the RNeasy Kit (Qiagen). PolyA^+ ^RNA preparation, reverse transcription using an oligo(dT) linker primer, second strand cDNA synthesis and library construction by ligating the cDNAs into a pBSIISK^+ ^vector via *Eco*RI and *Bam*HI sites was performed by vertis Biotechnologie AG (Freising, Germany). Two libraries were constructed and transformed into competent *E. coli *cells: library N0 (1920 clones sequenced) and a normalized library (library N1; 13824 clones sequenced). Normalization was performed by one cycle of denaturation and reassociation of the cDNAs in order to reduce the representation of highly abundant transcripts and to enhance the representation of low abundance transcripts. 83% of the transcripts were 0.8 to 1.6 kb in length. We tested for sequence information saturation by clustering the last 2 × 96 sequences produced with the already clustered sequences (see paragraph "Clustering"). This procedure showed that only 5 and 7 sequences, respectively, added new clusters to the database. Thus, to get only 10 % more sequence information, more than a doubling of the EST data would have been required.

DNA of individual clones was extracted using the magnetic bead technology on a Qiagen robot.

Custom primers and the cycle sequencing method were used for sequencing. Since sequences generated from the 3' direction (the polymerase synthesizes the polyA tails before it accesses the informative sequences) are of considerable lower quality than those generated from the 5' end, we decided to only produce 5'end derived sequences. The sequencing reaction products were separated on ABI3700 96 capillary machines. Quality clipping and vector removal was performed using phred.

### Clustering

The gap assembler implemented in the Staden package [41] enabled us to obtain a minimal contig set. Previous results have shown that an automated clustering alone does not join all possible overlaps due to accumulating sequencing errors in the end region of the sequences. This is particularly the case for contigs with low coverage. Since the joining of all truly overlapping sequences and disentanglement of wrongly joined sequences is a crucial step in the analysis of EST data, all contigs were manually inspected and edited. In cases where contigs overlapped with identical sequences but aligned only with introduced gaps we fused these contigs and treated them as potentially alternatively spliced. Single pass EST sequences are publicly available via dbEST [dbEST: : EL563888 to EL579567].

### Analysis

The whole data set was tested for contamination by applying blastn against the entire GenBank database with default values. This analysis revealed that only one single read had a good similarity on DNA level with already sequenced genomes [dbEST:EL577893]. Therefore we conclude that contamination with nucleic acids other than from *P. polycephalum *is probably on a very low level. However, we can not test and exclude that a few reads are derived from unsequenced genomes.

All sequences were compared as 6-frame translation of each EST to the protein databases Swissprot (swissprot version 54) and TrEMBL (uniprot_trembl version 37) using blastx. Blast parameters used (WUBlast2.0):

B 120 maximum number of database sequences for which *any *alignments will be reported

E 1E-15 the expectation threshold for reporting database hits

W 11 seed word length for the ungapped BLAST algorithm.

Furthermore, we used subdatabases of the refseq database to pinpoint the potential phylogenetic distribution of blast matches (refseq protein subdivisions for mammalia, other vertebrates, invertebrates, plants, protozoa, and microbes, generated 29^th ^March 2007). According to the occurring BLAST matches the contigs were binned into several categories (see table 2). This evolutionary categorization was repeated using a score threshold of 150 for all databases. The categorization does not take into account fungi specific gene matches. Thus, genes categorized to metazoa may also have matches to fungi-specific genes. Additionally, we created a database of all *D. discoideum *proteins and used it to determine the amoebozoa specific gene complement.

Six-frame translations were used to screen for Interpro domains [42]. The resulting domains were clustered according to categories shown in Fig. 1. Pathway analysis was done querying the KEGG database with the KAAS server [43].

## Competing interests

The author(s) declares that there are no competing interests. 

## Authors' contributions

GGl and WM conceived the study. GGl wrote the manuscript. SM searched for receptors and calculated the phylogenetic tree. GW and GGo contributed input on the analysis and on the improvement of the manuscript. All authors read and approved the final manuscript version.

## Supplementary Material

Additional File 1 Blast summary table. Blast summary table for all EST contigs including additional information on number of ESTs, EST IDs, alternative splicing, Interpro domains. Click here for file

Additional File 2 Signal transduction and cell cycle pathways. KEGG assignments of EST contigs for signal transduction and cell cycle. Click here for file

Additional File 3 Summary of interpro domain hits for the assembled contigs. A summary table for domains in all EST clusters detected via interproscan. Click here for file

Additional File 4 Alignments. Example alignments of alternatively spliced transcripts with genomic DNA. Click here for file

Additional File 5 Intron sequences determined. Sequences of introns used for the calculation of the splice site signatures and description of corresponding genes. Click here for file
