# Supplementary material for: A first glimpse at the transcriptome of Physarum polycephalum
Source: BMC Genomics. 2008 Jan 7;9:6. doi: 10.1186/1471-2164-9-6 (PMC2258281; doi:10.1186/1471-2164-9-6)
Supplement: Additional File 3 — Summary of interpro domain hits for the assembled contigs. A summary table for domains in all EST clusters detected via interproscan. [file 1471-2164-9-6-S3.doc]

Table S1: Summary of Interpro domains found in the EST contigs

| Number of individual Interpro IDs | 1111 |
| --- | --- |
| Contigs with Interpro hits | 2914 |
| Number of hits in same frame | 4492 |
| Number of genes with domain but without database match | 490 |
| All hits | 11091 |
