# Supplementary material for: A first glimpse at the transcriptome of Physarum polycephalum
Source: BMC Genomics. 2008 Jan 7;9:6. doi: 10.1186/1471-2164-9-6 (PMC2258281; doi:10.1186/1471-2164-9-6)
Supplement: Additional File 5 — Intron sequences determined. Sequences of introns used for the calculation of the splice site signatures and description of corresponding genes. [file 1471-2164-9-6-S5.doc]

Table S2: A Intron sequences determined. Sequences include bases from the adjacent exons. The splice sites are indicated via an underscore

| >gene1_A alternative acceptor 5 bp | GAAATCAATG_GtATTTAATATCATGCCTTAAGTAACAGTTTAATTCCTTAATCCCCAAATTCAAAGCTAATTTTTTTAAG_GGCAG_CAATAAC |
| --- | --- |
| >gene1_B intron retention + alternative acceptor 24 bp | TATAAATAACGAG_GTATAGCAACTATCGTGTGGTTCTCCTCCTCCCATACCAAGTATCTTATTCATTCACTTTTTTTGAAAAATTATTACAG_AGCTTTATTCCTGGATATTACCTAG_ATTTTGT |
| >gene1_C intron retention + alternative donor 40 bp | CTGCTTATTCTCA_GTATTCTGAGCTAGTTCAATATGTTTTACAGAACTATAGT_GTACGTTGCAATTAGTTTGTTGTTTTATTTTTACTTTGAAAATTTAATTTTCGAAGTTTATAATTTTATTTAATTTGCTTCTTTACGTACTATTTATTTGTGTATCATTTTATTTCTGTTTGTTATTTTTATCAAGTTTTTTGTTCTGTTTTAATTTCCAAATTTCAGTGCCTAATTGACGTAATTATTTAG_ATACCACAAATATC |
| >gene2_A intron retention | GCCAGTTTATATGG_GAAGGTTATTTTAAACATAATATAAAGACTGTTCATTTCCTTACAAGAATACAATACCCGTTAGGCAATATGACATTTTTTAAATATTTAAAAGAGTTTCCAAAATGAGCGTAGATATGTGGATAGTATCTTATACATAATTTTATTTTTTATTAGTTATTGTACAATAAGGTTTCTTTTAG_GACCACGGCA |
| >gene2_B intron retention | GTTCATCGAAGCAT_GTATCCTGCCATTAGTTTGTAACAAACAAACAAACAAACAAACAAACAACAACAACAACAAAAAAAAAAAAAAAAAGAGAGAAATAAAAAAACCAGGGGGGTACCACACAAGAAACAGTTTTTTATTTGACATGCGAAG_ACATGGATGGGCAACCTAAGC |
| >gene2_C intron retention | ACTGATATCAGATG_GTATGTACCTTTCGCTTTCCCTTTTACCATTTCGAAAGAAGGTAGTAAAGAAGAGGAAGATAGGGAGAAAGAGAGGGGCGATTGACACATCTGTCACAGG_GTAAATAAAGGCG |
| >gene2_D intron retention | ACTCCAA_GTATCCCTATTCTTTTTTTTTTCTGAAAAAGTTAAGCACAAATAAAATAACCTGGATCGTGTAGAATTACGCAAATATTACACAATTCTTTTTTGGAGTACATTTTTGATATAACCAAG_GTAAAAACAG |
| >gene2_E intron retention | GGGCATGATGGG_GTATGACTTCGTACTATTAATTTGAAAATAAAATAAAATAATAAAATAAAATGCTTAAAAAACAAAACTTAAATAGCAATTGGTTATTGAAATAAAACCTCAATTTTATGAGATTTGGCTCATTTAAGCTAACTTTACTGCCAG_GTGGAAATTGAGATGC |
| >gene2_F intron retention | GGCTATTCAACGACATG_GTATGTTATCTTAAAAAATAACCTTTGATTTCCGATCCTGACTTTTTGAAATTTGCAAAATTCGTGTAACATCCACCAAGAAATACAATCACCCATAGCTATTTGAAAGTTCATTACATCATTTACGCATCCTAAGGAAAAAAGAAAAATATTAGTATCTCTTTTAACTGCTTGATTACAG_AGCTGGCTGCTGGAAG |
| >gene3_A alternative donor 4 bp | TGCTCGTTTTGCAC_GTAT_GTACGTAACCATGTCCTCTTTACATTACGCAGTGTTGTTTCATTAACATCAAGTCACAG_ATACAGCTTCC |
| >gene3_B normal intron | CAACCCACTG_GTATGTCCTTTTCGTGATTGATTTATGATGTAAAGATCTTGTGTTCATCACCCAGGCCTATTGCTTTGTTGATAGTATTTGTTATTATTGTTTCTCATACTAGATGATTGTGTCCTTGTTCTTGCAGTGTAGATCTTTATGTCATTGGTCTTATGTGGTATTGATTCATGATTAG_ATTTTGATGATAAA |
| >gene3_C small exon inclusion (gene3_D second intron) | ATgaCaTTCACCcAAAtg_gTATTTTgaTcACCTTCATTCACACACCCCACAATCACATCACATCACATCACAgCACATCACATCACAgCACAgCACATCACATCACATCACATCACATCACATCACATCACATCACATCACATCACATCACATCACATCACATCACATCACATCACATCACATCACATCACATCACATCACATCACATCACATCACATCACATCACATCACATCACATCACATCCACTTATCTCTTGACCTTTGTCACTTCTACTTTTTAACAATTCTTGTTGTAG_GGGCCAAAGGCTCATTAGAG |
| >gene3_D small exon inclusion (gene3_C first intron) | CAACACAATG_GTATGCGTAACCTGTGGCTAGAATATAATTGAAACTAACTTAACATAACAG_ATCTATTACAAAAACAGA |
| >gene4_A normal intron | TATTGGGTGAAGAAaG_GTaCAAAGGATATAgaaaaCTACCGAAACCCCCCCAAATAGCGGAGAACGGAACTCCTTCAGAATCTAGCTTAACAAGGAAATTTATTGCATTGTGGCACTTTATAACCGAAAATATTTATGGCACACGAGATATTGCCCTAAACTGTTTTTGGTGCACACTTTACCACAAAAGTAAAACTTGCACTACAATTGCTGGTTTTGTTCAAAATAGCGCAAAATGCTGCGACAACATAAACTGTACTCTTTTATCATATTACAAATTGGTTATTAACTCTTAAAAGCACGCTCACATAACTTTATTTTGAATTTTCGTTGGTACGTAATGATGTTATGTCATAATTTTATCATTTACTATTCATCGAACCTAGCTACAACCATTAGTTACATAGATATTTGCATTGCGAAAAAATATTATTATGACAGCGCCACATTGACCATTCATAAGTAACTCCCATCCTTTAATTTTGTAAAAAATACTAGTAATTGCATTACACAACTCACCCAATTGATTGATCCGTTATAATTTCAAAATTAGAAAAAATTGATTAATTTACATTTTGATGCTAG_AATCCCAAGTAA |
| >gene4_B normal intron | gAACgTGAAAA_GTACGTAAaCTATTCTTTCaTTGAGCACTTCTAgAAATGtATTATTACTCCgtGAtGtGCtAATTGCGGTTATTGTAG_CTATTActGATGA |
| >gene4_C alternative acceptor 62 bp unknown intron structure | GCCACAAGGAGAGATGTTCGA_GTATCTTGCCATCCCTTCCCCTTAAATGTGTGTTAATTATTTCTTTTTTTGAACTTACAAAG_nnnnnnnTTTTTTTTTTTGCCTTGTACATATTTTTTATTAAAATCTACACAAATTTTGCTATAATATTTGCACTGTCTTACTCTGCTTAGCTTTTCCTACAACACTCACTGAAAATTTTTTACAAAAAATACATTATTTAGGTGTGATGTTGGACACAACAAGTTGAATCAACTTCAATGTTGATTCAACTTGATTGTTTTTTACTTACAAAG_ATTTTTACAGCTAGGACAACT |
| >gene4_D normal intron | ATaTTGCAGAATGG_GTAAGTTTTTAACAAATCAAATTACAATATTaTTTGGGGAAAAAAATTTTAAAAAAAACTAATTTTTTTTTTTTTTTTTTAAACTTAG_GTGAATGCCCTTAATGA |
| >gene4_E normal intron | TCGCTGGGACTACCAAAAG_GTATGCTGGGTCAGAAGTCAGAAACAAAAAAAGATACGTTCCGCTGTTTGATGAATATGTTATATTAAGTTTACTTTTCGTCGTGCAAATGGAGTGAATTCGATACGCCCATACAAGGTCCACCTAGTCCCATAATAAAAACAACGGCGATGGGAGAACAGCAATCGCCTGCAGACTGCGTACTAATGAGTTTTTCTAGTTCCACACGATTTGTCTTGAAATGAGCCATGAAATCCGATCCGGCCCACGCCACCCCAGCGAAGCATACCACAAGACAGAAGCAAGTGGAACACAAGAGGAGGCAATCGCGCGTTGACTGCGTACTAATCAGTTTTTTTATTAG_TCTACCACACTATT |
| >gene5_A intron retention | TTCTGTAGTTCTATA_GTCCGTACCTTGTTTTCTTCATATTCTTATTTATGTTACTTCTAGGCTCTAGTGAATTGGGATTCTTTCGTACTATTTCTATGCTTTTGATTCATTCACTTTGGATTCCCCCCCTTTTGCTGAATTTATCTTTTATTCCCTGCCATTCATCCTTGTTACTCTATTCTACATTCCACATATCTTTTCTCGATTTATGCACTCCTTCTCTTCTATATAATCATTCTAAATAATCATGTGCAG_GTAGTTTCGT |
| >gene5_B intron retention + alternative acceptor | CACAAGAGACAATAAATAAG_GTATTCATTTCTGTGTATATCTTGCATCTTTTCTCTTTCATCCTTGAAAGATTTTGGTGGGCATTGCTAGACCATTTCCACATCCACAGAGGAAATTAATTTTTGTATACATACATGTGTCTTCTTATTTCTTCTTTTGCGAGAAaaaaaaaaaaaaaaaaaaaaaaaaaCaAAAACCNAAAAgAAAAAGAAAGGCAATTCTAgAAAGGACTTCAGGGACTTTTTAAAaTTACGGAAGAAAAATTCTAATTTTCAaTATTGaAG_GTGTAagAGGCTTtCATAAAAAGGCACGTGgTCaTAATGTAGTTGAATGCCCAATATTATTTGTTTACTACAAAAGATCTACATCCCTTTTTTAAAAAAGAAAAACAAACGAAAAAGAAAATAAGCAAAATAAATTAATTCTAATACATTTGAAG_GTAGCAGCAGGGTTTCATA |
| >gene6_A intron retention + alternative acceptor | AAGGGGGATTTTT_GTATGTTTCCCTCGACAATCTACCCTCACCCCTTTGTTCTTCGTTTTTATGTTTTGATCTTATGCACGTGATGCACCAAATCTTTTTTCATAAATCAATAATAATTCTCTTAAAACTTCAATTCAG_AGTACTCATTTTTTGTTTGTTGTTTGAATACCAG_ACCACAAGAGAT |
| >gene6_B intron retention | GGCAAACGCGTAAG_GTAGTTCGACACCTCTAAACGTCGGCAACCTACCCTCCTACTATTTCCCTCTCATGCCTTCTACTTTGGGTTGCGAGTAACGGAGTTCTAGAGATTCTTTTCCCCTTAGCCCTTCCCTAAAGCTCTTATCCCCTTATCTTAGTACACTCTTATTACTTAGCACATAATTTGTTGTTAACCACCCCAACCACAACAG_TCAGCCATAA |
| >gene7_A normal intron | CCTAGTTCTAATAAG_GTATTCCTACCTCTTGTATAACTAAAGAATTTATCTTTTGTTTCATCAACCAATGCTGTGATTTTTTTATTGATTTTTTTTTTTTTTTTTTAATTCGTTTCACAAATTTAAATTAGTGAGTTTTCTTTGGATTCGTTTTTAAAACACTCCAAAGAACCTGTATTTGTAAATTATACTCTTTCATTCTTTCCATCCGCCTTGACAGATACCTCTTAATACCTTCATTACTCTATTTTATTATCACATTTTGGTGAATCGTGCATCTTATTATTTTTTCCAATGATTTATTTATTTTTTGTAG_CATGGCAGATGATG |
| >gene7_B alternative donors and acceptors | TGTTTTTGCAG_GTTTGCATCTCAAAATAAAGAATATAAATAATTAAAATAAAAAATAAAAATAAAATAAGATACGTAAAAAACGATTTAGCAAACGAAAAACGACAGTATTGCACATTTTTATTTTGTCAATTTTTAGTTCTTTATAAAAATGTGCACAATAAAAATAAATAAAAATACAATAAGATAAGATACAATAAGCTACTGTTAAAAAAATAAATCTAGTAAATGAGAAAACTAGACATAACATAGCTGTAATAAATAATAG_AAGGGGCAAAA |
| >gene8_A long intron | GTGTATgaATGTTG_GTATGAGTaAACaAACAATAAAACAAAGAAGAAGGATAAAAAAATAGGGAAAAGAAGAAAGGGAGAAGGAAGAAGAAAGCAGACGAAAGGGAGAAAGGAGAAAGGAGAAAGGAAGAGAAAGGAGAAAGGAAGAGAAAGGAAGAGAAAGGAGAAAAAAGAAAAAAGAGAAAAGAGAAAAGAGAAATGAGAAAAGAGAAAAGAGAAACGAGAAAAGAGAAAAGAGAAAAGAGAAAAGAGAAACAAGAAAAGAGAAAAGAGAAAAGAGGAAAGAGAAACGAGAAAAGAGAAAAGAGAAAAGAGAAAAGAGAAAAGAGAAAAGAGAAAAGAGAAAAGAGAAAAGAGAAAAGAGAAAAGAGAAAAGAGAAAAGAGAAAAGAGAAAAGAGAAACAAGAAAAGAGAAAAGAGAAACGAGAAAAGAGAAAAGAGAAAAGAGAAAAGAGAAAAGAGAAAAGAGAAAAGAGAAAAGAGAAAAGAGAAAAGAGAAAAGAGAAAAGAGAAAAGAGAAAAGAGAAAAGAGGAGAAAGAGAAAGGAGGAGGGAGAAAGGAGAAAGGGGGAGAAAGGGGGAGAAAGGAGAAAGGGGGAGAAAGGAGAAAGGGGGAGAAAGGAGAAAGGGGAGAAAGGAGAAAGGGGGAGAAAGGAGAAAGGGGGAGAAAGGAGAAAGGGGGAGAAAGGAGAAAGAAGAGGGGAAAGGAGAAAGGAGAGGGGAAAGGAGAAAGGAGAGGGGAAAGGAGAAAGGAGAAAGGAGCAAGGAGAAAAAATGGAAGAAAAGGAGAAAAGGGGAAATGGAAGGAAAGGTAAGGTAAGGGAAGGGAAGGGAAGGGAAGGGAATGAAAGGAAAGGGAATGAAAGGAAAGCAAATGAAAGGAAAGGAAGAGAAAGGAAAGAAAAGGAAAGAAAAGGAGTACACAGGAAAAGGTAAATAGAAAGAGTTATAGGCTGTTATAAAAGAGACAAATATTAAATATTAAATATTAAGCTTTACTAAATTTATAG_GCCTTGTAAATCAAGCTAGACTA |
| >gene8_B U12 intron | AACCATCTTTTCCCTTCTGATC_ATATCTTGTCCATCTCTTTCCCTCCTTTCCTTCCCTTTCATACCCCTACTCTCTCTCTCTCTCACTCCTCTCATGTTGCTTTTTTATTTTTCTTGCCCACTCTTTTCCTTCTTTTCTAATTCTTTGATTGATTTGCAA_GGCTTATAAAAAATGCAAGAA |
| >gene8_C long intron | TTGGGTTTTGAGTTCCAG_GTACTCTTTTTTTACCTTTCCCACTCTCCCCTTACTTTTCCCTTCTTCCCTTCATACTCATTCTTTTCCCTTCTTGCCCTTCCCCTTCCCCTTCTTTCCCCCTCTTTTGTCCTCTTTCCCATGCTTTCCTTTCCCTTTTTCATCTTTTCTTTTCTTTCTTTTCTTTTTCTATTCTATTCTCTTTCTTCTTTTCTTTTCTTTCTTTTCTTTTCTTTTCTTTTCTTTTCTTTTCTTTCTTTTCTTTTCTTAATGACATTTAG_GATTCATATCATTACCACCCCACATG |
| >gene8_D normal intron | ACAAGAAAGAGATA_GTATCTAAAAAGTTCTgcTcCtTTTAGTCAGAAACTAAAATTTTATAGCAAGGTccAcGtttgCCTTCTGAAGTATAGTTGTTAGTTAGCCTGAGTATTACACCTTGTCTATATCATaT_ATTTGTATTC |
| >gene9_A alternative donor intron retention | GGGCCAGTATAGT_GTATCCATTAAGGAGTGGGTAGTGTAACGGTGGGCCTGTGAAGGACCTCCAAATAGAAGCCACACATGATTG_GTATTCGGATGCCTTTTGGCCCAATGGTTAAATCATCTTGTCTTCTTTACAG_GTAGACGTCTCG |
| >gene9_B normal intron | CATCAAGTGGAGTCAG_GTTTGTTCATAGTCTTGACATACTATTTCTTTCGTTAATCTTTTGAAAG_TGATGAATCAGCAGCATTT |
| >gene9_C U12 intron | TTGGAGAAG_ATATCCTTTTATTTAATATCCCATATACAAAAGTAAATTCTTTGATTACTATGGTAA_AAATTATATCAA |
| >gene9_D normal intron | TTTGCTTTGAATTTCAG_GTATTGGCATATTTCAAAGCTGTTTATCTTATTTCCATGTTCGCGCCCAAATTTTAAATCCTTATTTGATTTACTAG_GATCCATATCATTAT |

B description of genes from which intron sequences were derived

| gene1 | EL567878 | Extracellular calcium-sensing receptor precursor (CaSR) (Parathyroid Cell calcium-sensing receptor) |
| --- | --- | --- |
| gene2 | EL570054 | unknown |
| gene3 | EL566457 | unknown IPR001261 ArgE/dapE/ACY1/CPG2/yscS metallopeptidase activity |
| gene4 | EL565032 | Hypothetical protein present in Amoebozoa1 IPR004843 Metallophosphoesterase hydrolase activity (GO:0016787) |
| gene5 | EL568744 | 4-coumarate--CoA ligase 4 |
| gene6 | EL566228 | unknown |
| gene7 | EL565880 | unknown IPR012317 PARP, catalytic NAD+ ADP_ribosyltransferase activity (GO:0003950) nucleus (GO:0005634) |
| gene8 | EL567881 | unknown |
| gene9 | EL564198 | DNA ligase |
